# A comparative study on the occurrence, genetic characteristics, and factors associated with the distribution of *Listeria* species on cattle farms and beef abattoirs in Gauteng Province, South Africa

**DOI:** 10.1007/s11250-024-03934-y

**Published:** 2024-02-27

**Authors:** J. Gana, N. Gcebe, R. Moerane, Y. B. Ngoshe, T. Tshuma, K. Moabelo, A. A. Adesiyun

**Affiliations:** 1 Department of Production Animal Studies, Faculty of Veterinary Science, University of Pretoria, Private Bag X04, Onderstepoort, Pretoria, 0110 South Africa; 2 Department of Agricultural Education, Federal College of Education, P.M.B. 39, Kontagora, Niger State Nigeria; 3 Bacteriology Department, Onderstepoort Veterinary Research, Agricultural Research Council, Pretoria, South Africa; 4 Epidemiology Section, Department of Production Animal Studies, Epidemiology Section, University of Pretoria, Private Bag X04, Onderstepoort, Pretoria, 0110 South Africa; 5 Department of Paraclinical Sciences, School of Veterinary Medicine, Faculty of Medical Sciences, University of the West Indies, St. Augustine, Trinidad and Tobago

**Keywords:** *Listeria*, Serogroups, Virulence genes, Cattle farms, Abattoirs

## Abstract

These cross-sectional studies reported the occurrence, genetic characteristics, and factors associated with the distribution of *Listeria* species on cattle farms and beef abattoirs in Gauteng Province, South Africa. A total of 328 samples (faeces, feeds, silage, and drinking water) were collected from 23 cattle farms (communal, cow-calf, and feedlot), and 262 samples (faeces, carcass swabs, and effluents) from 8 beef abattoirs (low throughput and high throughput) were processed using standard bacteriological and molecular methods to detect *Listeria* species. The factors associated with the prevalence of *Listeria* species were investigated, and multiplex polymerase chain reaction (mPCR) was used to determine *Listeria* species, the pathogenic serogroups, and the carriage of eight virulence-associated genes by *Listeria monocytogenes*. The overall prevalence of *Listeria* species in cattle farms was 14.6%, comprising *Listeria innocua* (11.3%), *Listeria monocytogenes* (3.4%), *Listeria welshimeri* (0.0%) compared with 11.1%, comprising *Listeria innocua* (5.7%), *Listeria monocytogenes* (4.6%), *Listeria welshimeri* (0.8%) for beef abattoirs. Of the three variables (area, type of farm/abattoir, and sample type) investigated, only the sample types at abattoirs had a significant (*P* < 0.001) effect on the prevalence of *L. innocua* and *L. welshimeri.* The frequency of distribution of the serogroups based on 11 *L. monocytogenes* isolated from farms was 72.7% and 27.3% for the serogroup 1/2a-3a and 4b-4d-4e, respectively, while for the 12 *L. monocytogenes* isolates recovered from abattoirs, it was 25%, 8.3%, 50% and 16.7% for the serogroup 1/2a-3a, 1/2b-3b, 1/2c-3c, and 4b-4d-4e respectively *(P* < 0.05). All (100%) isolates of *L. monocytogenes* from the farms and abattoirs were positive for seven virulence genes (*hlyA, inlB, plcA, iap, inlA, inlC,* and *inlJ)*. The clinical and food safety significance of the findings cannot be ignored.

**Supplementary Information:**

The online version contains supplementary material available at 10.1007/s11250-024-03934-y.

## Introduction

*Listeria monocytogenes* is a major cause of ruminant listeriosis, even though it can infect various animal species. Listeriosis in ruminants can also be caused by *L. ivanovii*, which is non-pathogenic for other animal species and humans (Kaur and Balgir 2021). Cattle and other livestock are exposed to these pathogens through improperly produced silage, feeds, and contaminated water (Rodriguez et al. 2021). Some clinical manifestations of listeriosis in ruminant animals include encephalitis. Listeriosis in cattle has been reported in several countries, such as Latvia (Terentjeva et al. 2021), the USA (Nightingale et al. 2004), Ireland (Hilliard et al. 2018), Jordan (Obaidat et al. 2020), Spain (Hurtado et al. 2017), England (McLauchlin et al. 2020) and Nigeria (Chuku et al. 2019). The *Listeria* species have been isolated from slaughterhouses/abattoirs in China (Zhao et al. 2021), Japan (Takahashi et al. 2007), Turkey (Al et al. 2022), Belgium (Demaître et al. 2020) and Nigeria (Aiyedun et al. 2020). Regarding the beef chain and the epidemiology of *L. monocytogenes*, the organism has been reported to be present on cattle farms, including feedlots and cow-calf operations (Mohammed et al. 2010). Slaughter cattle at abattoirs have been shown to shed *L. monocytogenes* in their faeces, making this an important source of infection in the meat value chain. The sources *of L. monocytogenes* on cattle farms have been reported to be feeds, including spoilt silages, faeces, and farm environments (Palacios-Gorba et al. 2021). At the abattoirs, *L. monocytogenes* has been isolated pre-slaughter from the faeces, peri-anal areas, and skins and carcasses of cattle (Foerster et al. 2012; Zhao et al. 2021).

Listeriosis is an important emerging foodborne disease, causing life-threatening infections in humans, including abortion and stillbirth in pregnant women, septicemia, encephalitis, meningitis, gastroenteritis, and perinatal infections (Dhama et al. 2015). The foodborne bacteria, *Listeria. monocytogenes* and other *Listeria* species are highly adaptable pathogens that can persist in various environmental and food-related chains (NicAogáin and O’Byrne, 2016). It can acclimatize and live in extensive stressed conditions such as low water activity, temperature, and pH, making it problematic for producers who depend on these stresses for conservation (Chapin et al. 2014).

Some characteristics of *L. monocytogenes*, including their serogroups and their carriage of virulence genes, have been associated with the ability of the microorganism to cause listeriosis. The serogroups of *L. monocytogenes* commonly detected on cattle farms, beef abattoirs, or slaughterhouses include 4b-4d-4e and 1/2b-3b (Castro et al. 2018), and 1/2a-3a, 1/2b-3b, 1/2c-3c and 4b-4d-4e (Demaître et al. 2021). These serogroups are of public health importance and are commonly associated with animal and human listeriosis cases. The predominant serogroups detected in listeriosis were 1/2a-3a, 1/2b-3b, 1/2c-3c, and 4b-4d-4e (Oh et al. 2018; Obaidat et al. 2020). Virulence genes, *hly*, *sigB*, *plcA*, *inlB*, *inlC actA*, *inlA*, *inlB*, *plcB*, *hlyA,* and *inlJ* have been documented in *L. monocytogenes* strains isolated from cattle farms, beef abattoirs and in human cases of listeriosis (Al et al. 2022; Oh et al. 2016; Pournajaf et al. 2016). However, the predominant virulence genes reported are *plcA*, *prfA*, *hlyA*, *inlB, inlA, inlC, inlJ, actA,* and *iap* (Kayode and Okoh 2022). Some of the modes of action of the prevalent virulence genes are the predominant activation of *hlyA* and *plcA* within the phagosomal compartment and *actA* and *inlC* in the host cell cytosol (Bubert et al. 1999), while *prfA* is activated by transcription of the listeriolysin gene (Chakraborty et al. 1992). Some of the virulence genes are involved in adherence to and internalization by the host cell (*inlA*, *inlB*, and *inlJ*), escape from the vacuoles (*hly*, *plcA*, and *plcB*), intracellular replication (*htp*), and cellular movement (*actA*) (Kastbjerg et al. 2010).

In South Africa, there is a dearth of information on epidemiological data on the samples assessed for contamination by *L. monocytogenes* and *Listeria* species, the risk posed to cattle, beef, and beef products, especially since ‘Polony’, a meat product, was responsible for the large listeriosis outbreak in the country; and the species of *Listeria*, other than *L. monocytogenes,* in beef and beef products (Allam et al. 2018). Recently, Matle et al. (2019) conducted a study on raw intact meat, ready-to-eat (RTE) meat products, and raw processed meat in the country’s nine provinces and reported a prevalence of 14.7% for *L. monocytogenes.* Therefore, the current study was conducted to determine the occurrence, genetic characteristics (pathogenic serogroups and virulence profiles), and the independent factors (area, type of cattle farms/abattoirs, and sample types) associated with the distribution of *Listeria* species on cattle farms and beef abattoirs in Gauteng Province, South Africa.

## Material and methods

### Study design and sample size determination

The cross-sectional study was conducted on 23 cattle farms and eight abattoirs in Gauteng province, South Africa, to determine the occurrence and characteristics of *Listeria* species in cattle farms and abattoirs. Gauteng province is one of the nine provinces and the smallest in South Africa, with approximately 15.81 million people.

A sample size of 328 and 262 for cattle farms and beef abattoirs, respectively, was determined using a formula by Thrusfield (2007); *n* = [1.96^2^ *P*_exp_ (1 − *P*_exp_)]/*d*^2^, where *n* = required minimum sample size, *P*_exp_ = estimated prevalence of listeriosis and *d* = desired absolute precision.

### Selection of cattle farms and beef abattoirs, sources, and types of samples, and transportation to the laboratory for processing

The study was designed to randomly select cattle farms and abattoirs from the list made available by the Gauteng Department of Agriculture and Rural Development and Environment (GDARDE). Once the owners and managers of cattle farms and beef abattoirs were unwilling to participate in the study because of the COVID-19 pandemic, the next farm on the list was selected by systematic random sampling.

For the cattle farms consisting of feedlots (*n* = 3), cow-calf operations (*n* = 10), and communal farms (*n* = 10) in Gauteng, South Africa, samples were aseptically collected as described by Onyeka et al. (2021). Faecal (rectal faecal grab or freshly voided faeces) of individual cattle, and environmental samples inclusive of pooled faeces from areas where cattle congregated, drinking water in troughs, feeds (grains and grass), and silage in feeding troughs.

At the beef abattoirs consisting of high throughput, HT (*n* = 6), and low throughput, LT (*n* = 2), the following samples were collected using the procedure described by Zweifel et al. (2005). Carcass sampling was done according to the European Union Decision 2001/471/EC. Swab samples were obtained from vertical and horizontal streaks by applying gentle pressure using a swab rinse kit (SRK) (Copan Diagnostics, Inc., UK). The sources and types of samples were as follows: Pre-slaughter faeces in the lairage, carcass swabs [pre-evisceration, post-evisceration, 24 h post-chilled carcasses (-4 °C—20 °C)], abattoir effluents (environmental).

All the cattle farms and beef abattoir samples were collected between 2020 and 2021.

The collected samples from the cattle farms and beef abattoirs were transported to the ARC-Onderstepoort Veterinary Institute’s Feed and Food Laboratory, ice-cooled within 12 h of collection, and processed within 48 h.

### Enrichment of samples collected from cattle farms and beef abattoirs and PCR detection of *Listeria* species

For faecal and swab samples, sterile spoons were used to scoop faecal samples (farm samples) from the cups to sterile Petri dishes to weigh 10 g of the faecal samples, which were transferred aseptically into stomacher bags that contained 90 ml ONE Broth-*Listeria* (Thermo Fisher, South Africa). The samples were homogenized (Stomacher Laboratory Blender 400, Seward Ltd., West Sussex, UK) at normal speed for 2 min, followed by 48 h aerobic incubation at 35˚ C. For the swab samples from abattoirs, we used a swab rinse kit (SRK) (Copan Diagnostics, Inc., UK), and one millilitre (1 ml) of sample in the SRK was removed into 90 ml tubes of ONE Broth-*Listeria* (Thermo Fisher, South Africa) for 48 h aerobic incubation at 35˚C. Feed samples were aseptically withdrawn from the cup using forceps, and 10 g of the feed samples (grass and grain) was weighed using a weighing balance and transferred into a stomacher bag which contained 90 ml of ONE Broth-*Listeria* (Thermo Fisher, South Africa), which was followed by homogenization and aerobic incubation at 35 ˚C for 48 h. The water centrifugation method was used to isolate *Listeria* species from water and effluent samples for drinking and effluent samples. For each sample, 100 ml was aliquoted into four 25 ml amounts in centrifuge bottles and then spun down at 15,493 × g for five minutes. The pellets were pooled from the four bottles and inoculated into 9 ml of ONE Broth-*Listeria* (Thermo Fisher, South Africa) for enrichment, followed by aerobic incubation at 35 ˚C for 48 h. The enriched broth was used to inoculate Brilliance-*Listeria* agar (BLA) (Thermo Fisher Scientific, South Africa) plates to isolate *Listeria* species. A loopful of enriched broth culture growth in ONE Broth-*Listeria* (Th Thermo Fisher, South Africa) was inoculated onto Brilliance *Listeria* Agar (BLA) plates and streaked for isolation. The inoculated plates were incubated aerobically at 35˚C for 48 h. Single colonies of suspected *Listeria* species (colonies that appeared blue without a halo) and *L. monocytogenes* (blue colonies with a white/cream halo) were phenotypically identified, as described by Jamali et al. (2013). PCR was used to confirm the isolates of *Listeria* species. DNA was extracted from enriched broth cultures and isolates by the boiling-centrifugation method, as described by Soumet et al. (1994). The DNA extracts used as templates in the mPCR assays were prepared as described by Soumet et al. (1994). All enriched broth samples were screened by multiplex PCR for *Listeria* species (*Listeria* genus), using the *prs* gene as a target marker (Supplementary Table S1). Screening by PCR was performed utilizing an mPCR assay that targets the genes listed in Supplementary Table S1, as described by Doumith et al. (2004). To detect the different species of *Listeria*, the DNA extracts used as templates in the mPCR assays were prepared as described by Soumet et al. (1994). The primers utilized in the current study are shown in Supplementary Table S2. The multiplex PCR mix was prepared as recommended (Doumith et al. 2004). PCR amplicons were electrophoresed on a 3.0% agarose gel using 1 × Tris–acetate-EDTA (TAE) buffer and stained with ethidium bromide (Ryu et al. 2013).

### Determination of the serogroups of *L. monocytogenes* isolates

The same mPCR assay method was used to detect the *Listeria* genus and to characterize *L. monocytogenes* regarding their serogroups. The mPCR targeting five gene fragments of *L. monocytogenes,* namely, *imo1118*, *imo0737*, *orf2110*, *orf2819*, and *prs* (specific for *Listeria* genus) was used to determine the serogroups of *L. monocytogenes* as previously described by Doumith et al. (2004). The five primers used to classify the strains into serogroups are shown in supplementary data, Table S1.

### Detection of virulence genes in *L. monocytogenes* isolates

The presence of selected virulence genes in the isolates of *L. monocytogenes* was determined as earlier described (Lomonaco et al. 2012; Pournajaf et al. 2016). Multiplex PCR was used to detect eight virulence-associated genes of *L. monocytogenes: plcA*, *hlyA*, *actA, inIB*, *lap, inlA*, *inlC,* and *inlJ* in two multiplexes. Multiplex 1 (mPCR 1) contained 5 primer sets (*plcA*, *hlyA*, *actA, inIB,* and *iap*). In comparison, Multiplex 2 (mPCR 2) consisted of three primer sets (*inlA*, *inlC,* and *inlJ*) (Supplementary Table S3).

### Data analysis

Laboratory data generated for the occurrence of the six species of *Listeria (L. monocytogenes, L. innocua, L. welshineri, L. grayi, L. ivanovii,* and *L. seeligeri)*, serogroups, and virulence-associated genes from beef and beef products collected at cattle farms and beef abattoirs in the current study were entered into Microsoft Excel 2016. The data were analyzed using Epi Info software (Version 7.0), and the association of variables (independent factors, e.g., area, type of farms and abattoirs, and types of samples) with the detection of *Listeria* or selected characteristics (dependent factors) was determined using Fishers Exact and Chi-square. The significant difference was evaluated using (P-value < 0.05), and percentages were calculated at a 95% confidence interval. Epi Info was also employed to generate percentages for categorical data on the prevalence of the six species of *Listeria* by the geographical location of farms and abattoirs, type of farms and abattoirs, and sample types.

## Results

### Occurrence of *Listeria* species on cattle farms and abattoirs

Overall, 14.6% (48/328) of the samples collected from the cattle farms were positive for the genus *Listeria,* while for the beef abattoirs, it was 11.1% (29/262). The difference was not statistically significant (*P* = 0.201). Of a total of 590 samples processed from the cattle farms and beef abattoirs, 77 (13.1%), 23 (3.9%), 52 (8.8%), and 2 (0.3%) were positive for *Listeria* species (*Listeria* isolates that could not be identified to species level), *L. monocytogenes*, *L. innocua,* and *L. welshimeri*, respectively (*P* < 0.001). *Listeria ivanovii, L. grayi,* and *L. seeligeri* were not detected in any of the samples from this study.

### Prevalence of *L. monocytogenes* on cattle farms and beef abattoirs and associated factors

The prevalence of *L. monocytogenes* on cattle farms and the univariate analysis of associated factors are shown in Table 1. The prevalence of *L. monocytogenes* was 3.4% (11/328). The three variables assessed (area, type of farm, and type of samples) had no statistically significant (*P* > 0.05) on the prevalence of *L. monocytogenes*. Table 2 shows the prevalence of *L. monocytogenes* in abattoirs and the univariate analysis of associated factors. The prevalence of *L. monocytogenes* on beef abattoirs was 4.6% (12/262). The location of the abattoirs, type of abattoir, and type of samples tested did not significantly (*P* > 0.05) affect the prevalence of *L. monocytogenes*. In comparison, although the prevalence of *L. monocytogenes* in samples collected from cattle farms, 3.4%, was lower than found in beef abattoirs, 4.6%, the difference was not statistically significant (*P* = 0.444).

**Table 1:** Prevalence of *Listeria monocytogenes* and *Listeria innocua* in samples collected from farms and univariate analysis of associated factors

			Prevalence (%) of:	
Variable	Level	No. of samples tested^a^	*L.monocytogenes*^*b*^	*L. innocua*^*c*^
Area	Winterveld	48	4.2	12.5
	Soshanguve	63	1.6	4.8
	Diepsloot Nature Reserve	16	6.3	6.3
	Acacia	15	0	13.3
	Doornrandjies	15	13.3	6.7
	Haakdooii-Gboom	13	0.0	15.4
	Hammanskrasl	15	6.7	13.3
	Moretele	45	2.2	13.3
	Cullinan	36	5.6	11.1
	Onderstepoort	31	3.2	12.9
	Bronkhorstspruit	31	0.0	19.4
	95%Cl		1.3–5.1	7.4–14.4
	*p-value*		0.50	0.811
Farm type	Communal	83	3.6	10.8
	Cow-calf	147	3.4	9.5
	Feedlot	98	3.1	14.3
	95%Cl		2.5–7.2	7.8–14.2
	*p-value*		0.196	0.508
Type of samples	Individual faeces	190	2.6	12.6
	Pooled faeces	75	2.7	6.7
	Feed-Grains/grass	27	11.1	3.7
	Silage	6	0.0	33.3
	Water	30	3.3	16.7
	95%Cl		1.4–5.3	8.1–15.1
	*p-value*		0.228	0.116

^a^328 No. of samples tested; ^b^3.4% of *L. monocytogenes;* ^c^11.3% of *L. innocua*

**Table 2:** Prevalence of *L. monocytogenes*, *L. innocua,* and *L. welshimeri* in samples collected from abattoirs and univariate analysis of associated factors

			Prevalence (%) of:		
Variable	Level	No. of samples tested^a^	*L. monocytogenes *^*b*^	*L. innocua *^*c*^	*L. welshineri*^*d*^
Area	Merafong	39	7.7	5.1	5.1
	Ekurhuleni	39	7.7	0.0	0.0
	Tswane	50	4.0	6.0	0.0
	Benoni	39	10.4	2.6	0.0
	Holfontein	26	0.0	3.8	0.0
	Cullinan	30	0.0	13.3	0.0
	Heidelberg	39	0.0	10.3	0.0
	95%Cl		2.0–7.1	2.9–8.6	0.3–1.8
	*p-value*		0.163	0.239	0.073
Abattoir type	High Throughput (HT)	216	5.6	5.6	0.9
	Low Throughput (LT)	46	0.0	6.5	0.0
	95%Cl		2.0–7.1	2.9–8.6	0.3–1.8
	*p-value*		0.102	0.07	0.429
Type of samples	Faecal Swab	66	1.5	7.6	0.0
	Pre-Evisceration Swab	66	7.6	4.5	0.0
	Post-Evisceration swab	66	4.5	0.0	0.0
	Chilled Swab	42	2.4	0.0	0.0
	Environmental Sample	22	9.1	31.8	9.1
	95%Cl		2.0–7.2	2.9–8.6	0.3–1.8
	*p-value*		0.355	< 0.001	< 0.001

^a^262 No. of samples tested; ^b^4.6% Prevalence of *L. monocytogenes*; ^c^5.7% Prevalence of *L. innocua; *^*d*^*0.8% Prevalence of L. welshineri*

### Prevalence of *L. innocua* on cattle farms and beef abattoirs and associated factors

The prevalence of *L. innocua* in cattle farms and the univariate analysis of associated factors are shown in Table 1. The overall prevalence of *L. innocua* on cattle farms was 11.3% (37/328). The three variables (area, type of abattoir, and type of samples) did not have a statistically significant (*P* > 0.05) effect on the prevalence of *L. innocua*. At the abattoirs, *L. innocua* was detected in 5.7% (15/262) of the samples processed (Table 2). Statistically significant (*P* < 0.001) differences were detected only among the type of samples tested, with a range from 0.0% (chilled carcass swabs) to 31.8% (environmental samples). Comparatively, the prevalence of *L. innocua* in the samples collected from cattle farms (11.5%) was statistically significantly higher (*P* = 0.018) than that detected in abattoir samples (5.7%).

### Prevalence of *L. welshimeri* in cattle farms and beef abattoirs

All the 328 samples collected from cattle farms were negative for *L. welshimeri*, with a prevalence of 0.0%. The prevalence of *L. welshimeri* in abattoirs and the univariate analysis of associated factors are shown in Table 2. The overall prevalence of the organism was 0.76% (2/262). The prevalence of *L. welshimeri* varied significantly (*P* < 0.001) across sample types only (environmental samples, 9.1% versus other types of samples, 0.0%). The difference between the prevalence of *L. welshimeri* on cattle farms (0%) and beef abattoirs (0.76%) was not statistically significant (*P* = 0.197).

### Frequency of the serogroups of *L. monocytogenes* isolated from cattle farms and beef abattoirs

The frequency distribution of the serogroups of *L. monocytogenes* isolated from farms was 72.7% (8/11) and 27.3% (3/11) for 1/2a-3a and 4b-4d-4e, respectively. The difference was statistically significant (*P* = 0.033). The distribution of the serogroups among isolates of *L. monocytogenes* recovered from cattle farms by area, type of farms, and type of samples is shown in Table 3. Statistically significant differences were detected in the frequency of *L. monocytogenes* serogroup 1/2a-3a according to the variables assessed as follows: area (*P* = 0.001), type of farm (*P* = 0.030) and type of sample (*P* = 0.030).

**Table 3:** Frequency of detection of *L. monocytogenes* serogroups by the area, farm type/size, and type of samples

	No. of isolates of *L. monocytogenes*	No (%) of isolates belonging to	No (%) of isolates belonging to
Variables		Serogroup 1/2a-3a	Serogroup 4b-4d-4e
Area			
Winterveld	2	2 (100.0)	0 (0.0)
Soshanguve	1	1 (100.0)	0 (0.0)
Diepsloot Nature Reserve	1	1 (100.0)	0 (0.0)
Doornrandjies	2	1 (50.0)	1 (50.0)
Hammanskraal	1	1 (100.0)	0 (0.0)
Moretele	1	0 (0.0)	1 (100.0)
Cullinan	2	1 (50.0)	1 (50.0)
Onderstepoort	1	1 (100.0)	0 (0.0)
*p-value*		0.001	0.104
Farm type			
Communal	3	3 (100.0)	0 (0.0)
Cow-calf	5	3 (60.0)	2 (40.0)
Feedlot	3	2 (66.7)	1 (33.3)
*p-value*		0.030	0.1869
Type of samples			
Individual faeces	5	3 (60.0)	2 (40.0)
Pooled faeces	2	2 (100.0)	0 (0.0)
Feed-Grains/grass	3	2 (66.7)	1 (33.3)
Water	1	1 (100.0)	0 (0.0)
*p-value*		0.020	0.180

For beef abattoir samples, the frequency of distribution of the serogroups among the *L. monocytogenes* isolates was 25% (3/12), 8.3% (1/12), 50% (6/12), and 16.7% (2/12) for the serogroup 1/2a-3a, 1/2b-3b, 1/2c-3c, and 4b-4d-4e respectively (Table 4). The area, type of farms, and type of samples did not significantly affect the serogroups of *L. monocytogenes* (*P* > 0.05).

**Table 4:** Frequency of detection of *L. monocytogenes* serogroups by the area, abattoirs type/size, and type of samples

	No. of isolates of *L. monocytogenes*	No (%) of isolates belonging to	No (%) of isolates belonging to	No (%) of isolates belonging to	No (%) of isolates belonging to
Variables		Serogroup 1/2a-3a	Serogroup 1/2b-3b	Serogroup 1/2c-3c	Serogroup 4b-4d-4e
Area					
Merafong	3	1 (33.3)	1 (33.3)	1 (33.3)	0 (0.0)
Ekurhuleni	3	2 (66.7)	0 (0.0)	0 (0.0)	1 (33.3)
Tswane	2	0 (0.0)	0 (0.0)	2 (100.0)	0 (0.0)
Benoni	4	0 (0.0)	0 (0.0)	3 (75.0)	1 (25.0)
*p-value*		0.215	0.391	0.100	0.188
Abattoir type					
High Throughput (HT)	12	3 (25.0)	1 (8.3)	6 (50.0)	2 (16.7)
*p-value*					
Type of samples					
Faecal Swab	1	0 (0.0)	1 (100.0)	0 (0.0)	0 (0.0)
Pre-Evisceration Swab	5	1 (20.0)	0 (0.0)	4 (80.0)	0 (0.0)
Post-Evisceration Swab	3	1 (33.3)	0 (0.0)	1 (33.3)	1 (33.3)
Chilled Swab	1	0 (0.0)	0 (0.0)	1 (100.0)	0 (0.0)
Environmental Sample	2	1 (50.0)	0 (0.0)	0 (0.0)	1 (50.0)
*p-value*		0.099	0.373	0.106	0.189

Among the serogroups of *L. monocytogenes*, statistically significant differences were detected in their frequencies between farm and abattoir isolates only for 1/2a-1/3a (*P* = 0.022) (72.7% versus 25%) and 1/2c-3c (*P* = 0.014) (0% versus 50%).

### Frequency of detection of virulence genes in *L. monocytogenes* isolates

For the 11 *L. monocytogenes* isolates from cattle farms and for the eight virulence genes assayed, seven genes *(hlyA, inlB, plcA, iap, inlA, nlC,* and *inlJ*), were detected in each of the isolates, 100% (11/11), while *actA* was detected in 9 of 110 (81.8%) isolates. Furthermore, of the total 8 farm isolates of *L. monocytogenes* that belonged to serogroup 1/2a-3a, only 6 (75%) were positive for virulence gene *actA* compared with the three isolates that belonged to serogroup 4b-4d-4e, which were all (100%) for the 8 virulence genes assayed, as shown in Table 5.

**Table 5:** Frequency of detection of *L. monocytogenes* virulence genes by the area, farm type/size, type of samples and serogroups

	No. of isolates of *L. monocytogenes*^*a*^	No (%) of isolates belonging to	No (%) of isolates belonging to	No (%) of isolates belonging to	No (%) of isolates belonging to	No (%) of isolates belonging to	No (%) of isolates belonging to	No (%) of isolates belonging to	No (%) of isolates belonging to	
Variables		Virulence gene hlyA	Virulence gene inlB	Virulence gene plcA	Virulence gene actA	Virulence gene iap	Virulence gene inlA	Virulence gene inlC	Virulence gene inlJ	*p*-value
Area										
Winterveld	2	2 (100.0)	2 (100.0)	2 (100.0)	1 (50.0)	2 (100.0)	2 (100.0)	2 (100.0)	2 (100.0)	** < 0.001**
Soshanguve	1	1 (100.0)	1 (100.0)	1 (100.0)	0 (0.0)	1 (100.0)	1 (100.0)	1 (100.0)	1 (100.0)	**0.0002**
Diepsloot Nature Reserve	1	1 (100.0)	1 (100.0)	1 (100.0)	1 (100.0)	1 (100.0)	1 (100.0)	1 (100.0)	1 (100.0)	
Doornrandjies	2	2 (100.0)	2 (100.0)	2 (100.0)	2 (100.0)	2 (100.0)	2 (100.0)	2 (100.0)	2 (100.0)	
Hammanskraal	1	1 (100.0)	1 (100.0)	1 (100.0)	1 (100.0)	1 (100.0)	1 (100.0)	1 (100.0)	1 (100.0)	
Moretele	1	1 (100.0)	1 (100.0)	1 (100.0)	1 (100.0)	1 (100.0)	1 (100.0)	1 (100.0)	1 (100.0)	
Cullinan	2	2 (100.0)	2 (100.0)	2 (100.0)	2 (100.0)	2 (100.0)	2 (100.0)	2 (100.0)	2 (100.0)	
Onderstepoort	1	1 (100.0)	1 (100.0)	1 (100.0)	1 (100.0)	1 (100.0)	1 (100.0)	1 (100.0)	1 (100.0)	
*p-value*					**0.00046**					
Farm type										
Communal	3	3 (100.0)	3 (100.0)	3 (100.0)	2 (66.7)	3 (100.0)	3 (100.0)	3 (100.0)	3 (100.0)	** < 0.001**
Cow-calf	5	3(100.0)	5 (100.0)	(100.0)	4 (80.0)	5 (100.0)	5 (100.0)	5 (100.0)	5 (100.0)	** < 0.001**
Feedlot	3	3 (100.0)	3 (100.0)	(100.0)	3 (100.0)	3 (100.0)	3 (100.0)	3 (100.0)	3 (100.0)	
*p-value*					**0.014**					
Type of samples										
Individual faeces	5	5 (100.0)	5 (100.0)	5 (100.0)	4 (80.0)	5 (100.0)	5 (100.0)	5 (100.0)	5 (100.0)	** < 0.001**
Pooled faeces	2	2 (100.0)	2 (100.0)	2 (100.0)	2 (100.0)	2 (100.0)	2 (100.0)	2 (100.0)	2 (100.0)	
Feed-Grains/grass	3	3 (100.0)	3 (100.0)	3 (100.0)	1 (33.3)	3 (100.0)	3 (100.0)	3 (100.0)	3 (100.0)	** < 0.001**
Water	1	1 (100.0)	1 (100.0)	1 (100.0)	1 (100.0)	1 (100.0)	1 (100.0)	1 (100.0)	1 (100.0)	
*p-value*					**0.016**					
Serogroup										
1/2a-3a	8	8 (100.0)	8 (100.0)	8 (100.0)	6 (75.0)	8 (100.0)	8 (100.0)	8 (100.0)	8 (100.0)	** < 0.001**
4b-4d-4e	3	3 (100.0)	3 (100.0)	3 (100.0)	3 (100.0)	3 (100.0)	3 (100.0)	3 (100.0)	3 (100.0)	
*p-value*					**0.09**					

^a^11 isolates of *L. monocytogenes* isolated from farm

Similarly, among the 12 isolates of *L. monocytogenes* recovered from abattoirs and for the eight virulence genes assayed, the frequency of seven genes *(hlyA, inlB, plcA, iap, inlA, nlC,* and *inlJ*) in each isolate was 100% (12/12), but for *actA,* the frequency was 83.3% (10/12).

Overall, the differences in the frequencies of virulence genes among isolates of *L. monocytogenes* recovered from cattle farms and beef abattoirs were not statistically significant (P > 0.05).

### Frequency of detection of virulence genes in *L. monocytogenes* isolates according to demography and serogroups

All the isolates of *L. monocytogenes* from the three types of farms (communal, cow-calf, and feedlot) were carriers of the seven virulence genes, but one isolate each from communal farms and cow-calf operation was negative for gene *actA*. A total of seven virulence genes were detected in the two serogroups (1/2a-3a and 4b-4d-4e), but the a*ctA* gene was detected in 75% (6/8) of the isolates in serogroup 1/2a-3a as shown Table 5.

For the *L. monocytogenes* isolates from beef abattoirs, all (100%) the isolates of *L*. *monocytogenes* were positive for seven genes *(hlyA, inlB, plcA, iap, inlA, nlC,* and *inlJ*) except for virulence gene, *actA*, detected in 10 (83.3%). The area, type of abattoir, and type of samples did not significantly (*P* > 0.05) affect the detection frequency of virulence-associated genes (Table 6).

**Table 6:** Frequency of detection of *L. monocytogenes* virulence genes by the area, abattoirs type/size, type of samples and serogroups

	No. of isolates of *L. monocytogenes*^*a*^	No (%) of isolates belonging to	No (%) of isolates belonging to	No (%) of isolates belonging to	No (%) of isolates belonging to	No (%) of isolates belonging to	No (%) of isolates belonging to	No (%) of isolates belonging to	No (%) of isolates belonging to	
Variables		Virulence gene hlyA	Virulence gene inlB	Virulence gene plcA	Virulence gene actA	Virulence gene iap	Virulence gene inlA	Virulence gene inlC	Virulence gene inlJ	*p-value*
Area										
Merafong	3	3 (100.0)	3 (100.0)	3 (100.0)	3 (100.0)	3 (100.0)	3 (100.0)	3 (100.0)	3 (100.0)	
Ekurhuleni	3	3 (100.0)	3 (100.0)	3 (100.0)	3 (100.0)	3 (100.0)	3 (100.0)	3 (100.0)	3 (100.0)	
Tswane	2	2 (100.0)	2 (100.0)	2 (100.0)	0 (0.0)	2 (100.0)	2 (100.0)	2 (100.0)	2 (100.0)	**0.0002**
Benoni	4	4 (100.0)	4 (100.0)	4 (100.0)	4 (100.0)	4 (100.0)	4 (100.0)	4 (100.0)	4 (100.0)	
*p-value*					**0.06**					
Abattoir type										
High Throughput (HT)	12	12 (100.0)	12 (100.0)	12 (100.0)	10 (83.30)	12 (100.0)	12 (100.0)	12 (100.0)	12 (100.0)	** < 0.001**
*p-value*										
Type of samples										
Faecal Swab	1	1 (100.0)	1 (100.0)	1 (100.0)	1 (100.0)	1 (100.0)	1 (100.0)	1 (100.0)	1 (100.0)	
Pre-Evisceration Swab	5	5 (100.0)	5 (100.0)	5 (100.0)	3 (60.0)	5 (100.0)	5 (100.0)	5 (100.0)	5 (100.0)	** < 0.001**
Post-Evisceration Swab	3	3 (100.0)	3 (100.0)	3 (100.0)	3 (100.0)	3 (100.0)	3 (100.0)	3 (100.0)	3 (100.0)	
Chilled Swab	1	1 (100.0)	1 (100.0)	1 (100.0)	1 (100.0)	1 (100.0)	1 (100.0)	1 (100.0)	1 (100.0)	
Environmental Sample	2	2 (100.0)	2 (100.0)	2 (100.0)	2 (100.0)	2 (100.0)	2 (100.0)	2 (100.0)	2 (100.0)	
*p-value*					**0.0003**					
Serogroups										
1/2a-3a	3	3 (100.0)	3 (100.0)	3 (100.0)	3 (100.0)	3 (100.0)	3 (100.0)	3 (100.0)	3 (100.0)	
1/2b-3b	1	1 (100.0)	1 (100.0)	1 (100.0)	1 (100.0)	1 (100.0)	1 (100.0)	1 (100.0)	1 (100.0)	
1/2c-3c	6	6 (100.0)	6 (100.0)	6 (100.0)	4 (66.7)	6 (100.0)	6 (100.0)	6 (100.0)	6 (100.0)	** < 0.001**
4b-4d-4e	2	2 (100.0)	2 (100.0)	2 (100.0)	2 (100.0)	2 (100.0)	2 (100.0)	2 (100.0)	2 (100.0)	
*p-value*					**0.002**					

^a^12 isolates of *L. monocytogenes* isolated from beef abattoir

For the total of six isolates of *L. monocytogenes* recovered from the abattoirs that belonged to three serogroups (1/2a-3a, 3 isolates; 1/2b-3b, 1 isolate, and 4b-4d-4e, 2 isolates), all (100%; 6/6) were positive for seven virulence genes (*(hlyA, inlB, plcA, iap, inlA, nlC,* and *inlJ*). However, for the six isolates that belonged to serogroup 1/2c-3c, only 4 (66.7%) were positive for virulence gene *actA* (Table 6).

Overall, there were no statistically significant differences (*P* > 0.05) in the frequencies of detection of virulence-associated genes in farm and abattoir isolates of *L. monocytogenes*.

## Discussion

For the first time, our study documented the prevalence and characteristics of *Listeria* species on cattle farms and beef abattoirs in South Africa. This is relevant and significant because the cattle farms (production sector) and beef abattoirs (processing sector) constitute parts of the beef production chain in the country, and ‘polony’, a ready-to-eat beef product, was implicated in the world’s largest known outbreak of human listeriosis, experienced by the country (Allam et al. 2018). Our report of *Listeria* prevalence and characteristics from the production and processing sectors adds to the prevalence data of *L. monocytogenes* in beef and beef products (retailing sector), which was earlier reported by Matle et al. (2019).

Although *L. monocytogenes* is the main species of *Listeria* responsible for livestock and human listeriosis (Hilliard et al. 2018; Koopmans et al. 2023), *L. ivanovii* has been associated primarily with livestock listeriosis (Arslan and Baytur 2019; Chand and Sadana 1999), and *L. innocua,* generally considered a non-pathogen*,* has been implicated in listeriosis in immunocompromised humans (Moura et al. 2019; Perrin et al. 2003). Hence, in the current study, these three Listeria species were investigated in addition to L. seeligeri and *L. grayi.* Of the five species of *Listeria* (*L. monocytogenes, L. innocua, L. seeligeri, L. ivanovii, L. grayi,* and *L. welshimeri)* investigated, only two (*L. monocytogenes* and *L. innocua*) were detected on the cattle farms. It is important that we detected *L. monocytogenes* in 3.4% of the samples collected from the cattle farms. This prevalence is higher than the 0.5% found on cattle farms in China but considerably lower than the range of 19% to 42.3% reported in other countries ( Nightingale et al. 2004; Obaidat and Stringer 2019; Hurtado et al. 2017).

In our study, the prevalence of *L. monocytogenes* according to the types of farms was low. It did not vary significantly across farms (communal farm: 3.4%, cow-calf: 3.4%, feedlot: 3.1%), which were slightly different from the findings of 3.1% and 0.3% reported in cow-calf and feedlots, respectively on cattle farms in central and southern California, USA (Mohammed et al. 2010); and was considerably lower than the farm prevalence of 11% reported in Latvia by Terentjeva et al. (2021). The low farm prevalence of *L. monocytogenes* found in our study is an indication that cattle farms in Gauteng province may not be important sources of *L. monocytogenes* to cause cattle listeriosis or to the contamination of slaughterhouses or abattoirs when the cattle are slaughtered.

Interestingly, all the silage samples processed in the current study were negative for *L. monocytogenes*, which may have contributed to the relatively low prevalence of the pathogen detected on the farms. It has been documented that silage, mainly if poorly fermented or of poor quality, harbour *Listeria* spp. (Mohammed et al. 2010; Rodriguez et al. 2021), and its consumption has been associated with livestock listeriosis, thus posing a threat to public health (Driehuis et al. 2018; Peng et al. 2022; Queiroz et al. 2018). The prevalences of *L. monocytogenes* in the other sample types reflect the carriage of the pathogen (pooled faeces: 2.7%) and the cattle’s risk of exposure to the pathogen through communal consumption of (feed, 11.1%) and water (3.3%) in troughs. Compared to our study, where the carriage and faecal shedding of *L. monocytogenes* was 2.7%, others have documented higher frequencies of 7.1% in Jordan (Obaidat et al. 2020), 18.2% in Slovenia (Bandelj et al. 2018), 28% in Latvia (Terentjeva et al. 2021**).** Similarly, there was a considerably higher prevalence of *L. monocytogenes* in mixed feed from the feeding troughs and hay (29%) and in drinking water troughs (28%) on cattle farms in Latvia by Terentjeva et al. (2021). Variability in the farm prevalence of *L. monocytogenes* may be partly explained by differences in the faecal shedding of the pathogen, the contamination of the feeds, drinking water, farm environments, and management practices (Ferreira et al. 2014; Hurtado et al. 2017; Stipetic et al. 2016; Terentjeva et al. 2021).

In our study, *L. innocua* was detected with a higher farm prevalence than *L. monocytogenes* (11.3% versus 3.4%) and from each sample type (faeces, feeds, silage, and drinking water) collected from cattle farms in our study. These findings agree with published reports that *L. innocua* has a broader distribution on cattle farms elsewhere (Gradovska et al. 2023; Gana et al. 2023). However, considering the organism is viewed as a non-pathogen, the risk of causing livestock and human listeriosis is minimal.

Unlike the farm samples, three *Listeria* species (*L. monocytogenes*, *L. innocua,* and *L. welshimeri*) were recovered from the abattoirs in our study at a prevalence of 4.6% (*L. monocytogenes*), 5.7% (*L. innocua*), 0.8% (*L. welshineri*). Compared with a similar study conducted in abattoirs in Jos, Nigeria, Dunka et al. (2021) reported a prevalence of 2.5%, 33.6%, 4.4%, and 1.7% for *L. monocytogenes*, *L. ivanovii*, *L. grayi*, and *L. seeligeri*, respectively. The prevalence of *L. monocytogenes* in the abattoir samples in the current study is considerably lower than reported for abattoirs in other countries (Al et al. 2022; Demaître et al. 2021).

The differences in the types and frequencies of *Listeria* species detected in abattoirs across countries may be partly due to the prevalence of *L. monocytogenes* in slaughtered cattle and sanitary practices during slaughter, which affect cross-contamination of carcasses by *L. monocytogenes* and other pathogens (Demaître et al. 2020; 2021; Mpundu et al. 2022a; Onyeka et al. 2021). Contrary to the lack of any significant effect of the three variables on the prevalence of *L. monocytogene*s in our study, others have documented the impact of the regional location of abattoirs (Demaître et al. 2021), type of abattoirs, HT versus LT (Onyeka et al. 2021), and types of samples (Dunka et al. 2021; Matle et al. 2019) on the prevalence of *L. monocytogenes.*

The comparatively slightly higher prevalence of *L. monocytogenes* detected at abattoirs (4.6%) than in cattle farms (3.4%) may be explained, in part, by the types of samples processed, which are exposed to a variable degree of cross-contamination. This is because, on cattle farms, the samples collected (silage, feces, feeds, water, and effluents) experience limited cross-contamination compared with abattoir samples (pre-slaughter faecal samples, pre- and post-evisceration swab samples, and chilled carcass swabs) which are subjected to cross-contamination. Cross-contamination of carcasses by *L. monocytogenes*, *Salmonella* spp., Shiga-toxin *Escherichia coli* (STEC), and other pathogens have been reported in the abattoir settings in South Africa and elsewhere (Manqele 2018; Onyeka et al. 2020; Rhoades et al. 2009).

*Listeria welshimeri* was isolated at a frequency of only 0.8% from beef abattoir samples in our study, a frequency considerably lower than reported by others, ranging from 3.8% to the 22% recorded in abattoirs in other countries (Al et al. 2022; Mpundu et al. 2022b; Mpundu et al. 2022b). Although *L. welshimeri* is being documented in abattoir samples for the first time in the country, it is generally considered non-pathogenic (Korsak and Szuplewska 2016).

It is of zoonotic significance that our study detected two pathogenic serogroups of *L. monocytogenes*, 1/2a-3a and 4b-4d-4e, in cattle farm isolates. Serogroup 1/2a-3a was predominantly detected (72.7%), which agrees with a study conducted in the USA, where 1/2a was the predominant serotype recovered from cattle farms (Borucki et al. 2005). The pathogenicity of serotype 1/2a has been attributed to its ability to form biofilms (Huang et al. 2018) and its high resistance to sanitizer and bacteriocins (Orsi et al. 2011). This is indicative that, although the overall prevalence of *L. monocytogenes* is low (3.4%), cattle exposed to the pathogenic serogroups of *L. monocytogenes* may be at risk of listeriosis. On the contrary, four pathogenic serogroups, 1/2a-3a, 1/2b-3b, 1/2c-3c, and 4b-4d-4e, were found in our abattoir isolates. These findings agree with the three serogroups (1/2a-3a, 1/2b-3b, and 4b-4d-4e) isolated from Belgian cattle slaughterhouses by Demaître et al. (2021) as these serogroups are commonly isolated from ruminants and widespread in the environment. Furthermore, Wieczorek et al. (2012) indicated that *L. monocytogenes* contaminated beef carcasses during the slaughter process predominantly harboured serotypes 1/2a, 1/2c, 4b, 1/2b, which also agree with the serogroups detected in the current study. It is also pertinent to mention that these serotypes are the dominant serotypes for food strains, accounting for human and ruminant listeriosis (Wang et al. 2015). Even though we did not classify the isolates into serotypes but, serogroups containing some of the known pathogenic serotypes, the majority harbored genes responsible for virulence in *L. monocytogenes*, highlighting the pathogenic potential of these isolates. Therefore, our findings of pathogenic serogroups on carcasses in the abattoirs sampled provide helpful information about the dominant serogroups of *L. monocytogenes* in beef abattoirs and their potential food safety implication in Gauteng province, South Africa, for policymaking, surveillance, and biosecurity. The detection of different serogroups and frequencies in the isolates of *L. monocytogenes* recovered from cattle farms and beef abattoirs in Gauteng province may be attributed to factors such as the fact that the study design is cross-sectional and not longitudinal, the samples collected and assessed at the farm level originated from only 23 farms in the country, which did not represent the origins of the cattle slaughtered at the eight abattoirs included in the current study.

It is of food safety and public health significance that all the 23 isolates of *L. monocytogenes* recovered from cattle farms and abattoirs in the current study were carriers of seven virulence genes *(hlyA, inlB, plcA, iap, inlA, inlC,* and *inlJ)*) while 19 (82.6%) were positive for the *actA* gene. This is because the pathogenicity of *L. monocytogenes* has been associated with the possession of virulence genes, especially those present in the *Listeria* Pathogenicity Islands (LIPIs) (Lopez-Valladares et al. 2018; Wiktorczyk-Kapischke et al. 2023). Three (*plcA, hlyA,* and a*ctA*) of the virulence genes detected in our study belong to the LIPI-1 cluster genes known to be involved in the infectious life cycle and survival in the food processing environment (Koopmans et al. 2023; Lopez-Valladares et al. 2018). Of relevance is the documentation that virulence genes, including those detected in our study, perform different roles and functions in the pathogenesis of *L. monocytogenes* and have been implicated in human listeriosis (Koopmans et al. 2023). The high frequency of the eight virulence genes assayed, and the detection of pathogenic serogroups in our *L. monocytogenes* may increase the pathogenicity of the *L. monocytogenes* we detected on cattle farms and abattoirs in our study. In agreement with our findings, Matle et al. (2019) detected the same eight virulence at similar frequencies from *L. monocytogenes* isolates from meat and meat products in South Africa. Varying types and frequencies of virulence genes have been reported for farm and abattoir isolates of *L. monocytogenes* in other countries (Ayaz et al. 2018; Obaidat et al. 2020; Wieczorek et al. 2012). Most virulence genes detected in our abattoir isolates of *L. monocytogenes* have been associated with human listeriosis (Arslan and Baytur 2019; Koopmans et al. 2023; Soni et al. 2015). The detected high frequency (83.3%-100.0%) of the eight virulence genes in pathogenic serogroups of the *L. monocytogenes* isolates recovered in the current study could pose a health risk to humans if contaminated beef and beef products from these sources are consumed.

## Conclusions and recommendations

For the first time, the current study demonstrated the presence and distribution of *L. monocytogenes*, *L. innoua,* and *L. welshimeri* in various sample types collected from cattle farms and beef abattoirs in South Africa. The prevalence of *L. monocytogenes* in samples collected at both the farm (production industry) and abattoir (processing industry) of the beef chain has food safety and public health significance because they belong to pathogenic serogroups and carry virulence genes associated with ruminant and human listeriosis. The detection of *L. innocua* from cattle farms and abattoirs indicates contamination. It can potentially cause listeriosis in immunocompromised humans should the strains enter the food chain in the farm-abattoir sector of the human food chain.

It is recommended that the contamination of feed and water at the farm level and carcasses of slaughtered animals by *L. monocytogenes* be reduced through good sanitary practices at both levels to prevent the entry of the pathogen into the human food chain. It is also recommended that a comprehensive risk assessment of the three variables (area, type/size of farm/abattoir, and sample types) investigated in the current study and other variables be conducted using a higher number of farms and abattoirs across the country’s eight provinces, to determine their importance as risk factors for the occurrents of *L. monocytogenes* and other *Listeria* species on cattle farms and abattoirs, in the country.

### Supplementary Information

Below is the link to the electronic supplementary material.

Supplementary file1 (DOCX 31 KB)

## Data Availability

All the data are contained within the article.
