# Supplementary material for: A comparative study on the occurrence, genetic characteristics, and factors associated with the distribution of Listeria species on cattle farms and beef abattoirs in Gauteng Province, South Africa
Source: Trop Anim Health Prod. 2024 Feb 27;56(2):88. doi: 10.1007/s11250-024-03934-y (PMC10896870; doi:10.1007/s11250-024-03934-y)
Supplement: Supplementary file 1 — Supplementary file1 (DOCX 31 KB) [file 11250_2024_3934_MOESM1_ESM.docx]

**Supplementary data, Table S1, S2, and S3**

Table S1. Primers used for mPCR to determine the presence of *Listeria* genus, and serogroup *L. monocytogenes in* the study (Doumith et al., 2004)

| PCR assay | Target Gene | Product Size (bp) | Primer Sequences (5′‐3’) |
| --- | --- | --- | --- |
| mPCR1 | *ORF2110* | 597 | *ORF2110*‐F:  *AGTGGACAATTGATTGGTGAA* |
|  |  |  | *ORF2110*‐R: *CATCCATCCCTTACTTTGGAC* |
|  | *ORF2819* | 471 | *ORF2819*‐F: *AGCAAAATGCCAAAACTCGT* |
|  |  |  | *ORF2819*‐R: *CATCACTAAAGCCTCCCATTG* |
|  | *Imo1118* | 691 | *lmo1118*‐F: *AGGGGTCTTAAATCCTGGAA* |
|  |  |  | *Imo1118*‐R: *CGGCTTGTTCGGCATACTTA* |
|  | *Imo0737* | 906 | *lmo0737*‐F: *AGGGCTTCAAGGACTTACCC* |
|  |  |  | *lmo0737*‐R: *ACGATTTCTGCTTGCCATTC* |
|  | *Prs* | 370 | *prs*‐ F: GCTGAAGAGATTGCGAAAGAAG |
|  |  |  | *prs‐R: CAAAGAAACCTTGGATTTGCGG* |
|  |  |  |  |

Table S2. Primers used for mPCR speciation in this study (Ryu et al., 2013)

| Species | Gene | Primer | Sequences (5^i^-3^i^) | PCR Product Size (bp) | Primer concentration (mM) |
| --- | --- | --- | --- | --- | --- |
| *Listeria* genus | *Prs* | *prs-F* | GCTGAAGAGATTGCGAAAGAAG | 370 | 0.2 |
|  |  | *prs-R* | CAAAGAAACCTTGGATTTGCGG |  |  |
| *L.* *grayi* | *Oxidoreductase* | *JOgrayi-F* | GCGGATAAAGGTGTTCGGGTCAA | 201 | 0.24 |
|  |  | *JOgrayi-R* | ATTTGCTATCGTCCGAGGCTAGG |  |  |
| *L.* *innocua* | *lin0464* | *lin0464-F* | CGCATTTATCGCCAAAACTC | 749 | 1.2 |
|  |  | *lin0464-R* | TCGTGACATAGACGCGATTG |  |  |
| *L. ivanovii* | *namA* | *liv22-228-F* | CGAATTCCTTATTCACTTGAGC | 463 | 0.52 |
|  |  | *liv22-228-R* | GGTGCTGCGAACTTAACTCA |  |  |
| *L. monocytogenes* | *Imo1030* | *lmo1030-F* | GCTTGTATTCACTTGGATTTGTCTGG | 509 | 0.56 |
|  |  | *lmo1030-R* | ACCATCCGCATATCTCAGCCAACT |  |  |
| *L.* *seeligeri* | *lmo033* | *lseelin-F* | GTACCTGCTGGGAGTACATA | 673 | 0.96 |
|  |  | *lseelin-R* | CTGTCTCCATATCCGTACAG |  |  |
| *L.* *welshimeri* | *scrA* | *lwe1801-F* | CGTGGCACAATAGCAATCTG |  |  |
|  |  | *lwe1801-R* | GACATGCCTGCTGAACTAGA | 281 | 0.96 |

| Table S3. Primer sequences, PCR preparation, and PCR condition used for virulence gene detection in this study | | | | | | |
| --- | --- | --- | --- | --- | --- | --- |
|  |  |  |  |  |  |  |
| PCR | Target | Product |  |  |  |  |
| assay | Gene | Size (bp) | Primer Sequences (5^’^ 3’) | PCR Preparation | PCR Condition | References |
|  |  |  |  |  |  |  |
|  |  |  |  |  |  |  |
| mPCR1 | *inlB* | 376 | *inlB*‐F: *GATATTGTGCCACTTTCAGGTT* | 12.5 μL 2× *DreamTaq* master mix, | 2 min at 94°C, | Liu et al. (2007) |
|  |  |  | *inlB*‐R: CCTCTTTCAGTGGTTGGGTT | 5 μL nuclease-free water, | 35 cycles of 94°C for 30 s, |  |
|  | *plcA* | 1484 | *plcA*‐F: CTGCTTGAGCGTTCATGTCTCATCC*C* | 5 μL template DNA, | 55°C for 30 s, |  |
|  |  |  | *plcA*‐R: ATGGGTTTCACTCTCCTTCTAC | 3 μL primer mix for mPCR2 | 72°C for 1 min and |  |
|  | *hlyA* | 456 | *hly*A*‐*F: GTTAATGAACCTACAAGACCTTCC |  | a final extension at 72°C for 10 min. |  |
|  |  |  | *hly*A*‐*R: ACCGTTCTCCACCATTCCCA |  |  |  |
|  | *actA* | 839 | *actA*‐F: TCGCCGCGGAAATTAAAAAAAGA |  |  |  |
|  |  |  | *actA*‐R: ACGAAGGAACCGGGCTGCTAG |  |  |  |
|  | *Iap* | 131 | *iap*‐F: ACAAGCTGCACCTGTTGCAG |  |  |  |
|  |  |  | *iap*‐R: TGACAGCGTGTGTAGTAGCA |  |  |  |
| mPCR2 | *inlA* | 800 | *inlA*‐F: *ACGAGTAACGGGACAAATGC* |  |  | Liu et al. (2007) |
|  |  |  | *inlA*‐R: CCCGACAGTGGTGCTAGATT |  |  |  |
|  | *inlC* | 517 | *inlC*‐F: AATTCCCACAGGACACAACC |  |  |  |
|  |  |  | i*nlC‐R:*CGGGAATGCAATTTTTCACTA |  |  |  |
|  | *inlJ* | 238 | *inlJ*‐F: TGTAACCCCGCTTACACACAGTT |  |  |  |
|  |  |  | *inlJ‐R:* AGCGGCTTGGCAGTCTAATA |  |  |  |
